# Trends in medical care utilization in patients with cancer: An analysis of real‐world data in a tertiary hospital in Korea, 2014–2019

**DOI:** 10.1002/cam4.6660

**Published:** 2023-10-30

**Authors:** Jung‐Hyun Won, Tae Kyu Chung, Joochul Lee, Sangwon Yoon, Yoomin Jeon, Howard Lee

**Affiliations:** ^1^ Department of Molecular Medicine and Biopharmaceutical Sciences, Graduate School of Convergence Science and Technology Seoul National University Seoul Korea; ^2^ Center for Convergence Approaches in Drug Development, Graduate School of Convergence Science and Technology Seoul National University Seoul Korea; ^3^ Department of Applied Bioengineering, Graduate School of Convergence Science and Technology Seoul National University Seoul South Korea; ^4^ SELVAS AI Inc. Seoul South Korea; ^5^ Department of Clinical Pharmacology and Therapeutics Seoul National University College of Medicine Seoul Korea; ^6^ Advanced Institute of Convergence Technology Suwon Korea

**Keywords:** cancer, immune‐oncology agent, IMRT, medical care utilization, targeted agent

## Abstract

**Background:**

Rising costs of cancer treatments challenge even areas with universal health coverage. There's a need to assess current medical care utilization trends among patients with cancer to guide public health policy, resource allocation, and set informed healthcare goals.

**Methods:**

We analyzed the latest trends in medical care utilization by cancer patients in four areas—drugs, radiation therapy (RT), surgery, and diagnostic procedures—using clinical databases extracted from electronic medical records of a tertiary hospital in Korea between 2014 and 2019. Compound adjusted growth rates (CAGR) were computed to capture the annual growth over the study period.

**Results:**

A total of 74,285 cancer patients were identified, with 40.3% (29,962), 14.2% (10,577), 31.1% (23,066), and 92.6% (68,849) of patients having received at least one anticancer agent, RT, surgery, and diagnostic procedure, respectively, over the period. We observed a 1.7‐fold increase in the use of targeted · immune‐oncology agents (from 6.8% to 11.6%) and a 21‐fold increase (from 3.0% in 2014 to 65.7%) in intensity‐modulated RT (IMRT) use over the period. In contrast, we observed a continuous decrease in the proportion of patients who underwent surgical treatment from 12.2% in 2014 to 10.9% in 2019. This decrease was particularly noticeable in patients with colon cancer (from 28.5% to 24.2%) and liver cancer (from 4.1% to 2.9%).

**Conclusion:**

From 2014 to 2019, there was a significant rise in the use of targeted · immune‐oncology agents and IMRT, alongside a decline in surgeries. While targeted · immune‐oncology agents and IMRT may offer promising outcomes, their financial impact and potential for overuse necessitate careful oversight and long‐term cost‐effectiveness studies.

## 1 INTRODUCTION

As the number of cancer treatment modalities has increased and their types have been diversified over the last few decades, the costs have also dramatically increased.^1^ For example, the annual price of novel anticancer agents easily exceeds 100,000 US dollars (USD).^2^, ^3^ Furthermore, innovative treatments such as CAR T‐cell therapy can cost >500,000 USD per year.^4^ Besides, the advent of advanced diagnostic techniques (e.g., liquid biopsy and multiparameter genomic assays) have further increased the cost of cancer treatments.^5^, ^6^, ^7^ Notably, in South Korea, cancer treatment expenses escalated by approximately 50 billion Korean won (approximately >37,000,000 USD) from 2014 to 2019, with an average annual growth rate of 12.7%.^8^

Thus, many cancer patients may not afford to receive optimal treatments because of their high costs. For example, patients with cancer often take less than the prescribed dose, and avoid filling prescriptions because of the financial burden.^9^ Furthermore, increased out‐of‐pocket expenses for cancer treatment inhibit patients from getting access to optimal medical care irrespective of age and insurance status.^1^

Universal health coverage, as exemplified by South Korea, aims to ensure all individuals have access to essential healthcare without financial strain.^10^ However, South Korea's universal health coverage does not cover all medical services.^8^, ^10^ In South Korea, the expenses for these non‐covered medical services have consistently risen, with an average annual growth rate of 7.7% over the past 5 years, from 11.5% in 2015 to 16.6% in 2019.^8^, ^11^ Among the four major diseases—cancers, cerebrovascular diseases, cardiac diseases, and rare intractable diseases—cancer treatment expenses emerge as the most significant concern, accounting for 11.0% of costs not covered by insurance, surpassing cerebrovascular diseases (5.1%), cardiac diseases (5.2%), and rare intractable diseases (2.7%).^11^

Despite the existence of South Korea's national health insurance, patients still face significant out‐of‐pocket costs, which affects the quality of cancer care.^12^ This underscores the urgency of smartly allocating limited resources. Real‐world data (RWD) has been essential in determining these priorities, guiding decision‐making, resource allocation, and setting healthcare objectives based on current cancer treatment patterns. For example, studies have shown that the use of intensity‐modulated RT (IMRT) and targeted therapies have increased to treat solid tumors.^13^, ^14^, ^15^, ^16^, ^17^, ^18^ Previous studies also have identified age‐specific clinical practices for cancer treatments. For example, hormonal therapy was the treatment of choice in elderly breast cancer (BC) patients aged ≥75 years, while chemotherapy was more frequently prescribed in younger patients.^19^

Indeed, those studies have provided insights into how to better shape public health policy for patients with cancer based on the contemporary treatment trends.^20^ However, those previous studies have been limited in that they did not include treatments that were newly developed and/or not covered by insurance. Furthermore, few studies comprehensively analyzed the latest trends in the treatments and diagnostic procedures for patients with cancer.^21^, ^22^, ^23^, ^24^

Based on this understanding, the objective of this study was to analyze the latest trends in medical care utilization by patients with cancer in the following four areas: drugs, RT, surgery, and diagnostic procedures. To this end, we used two clinical databases extracted from electronic medical records (EMR) of a university hospital for recent 6 years.

## 2 MATERIALS AND METHODS

### 2.1 Data sources and ethical statement

We used EMR data extracted, transformed, and loaded (i.e., ETL process) according to the common data model (CDM) specifications by the Observational Medical Outcomes Partnership (version 5.3) and the clinical data warehouse or CDW (SUPREME®) at Seoul National University Hospital (SNUH), a university‐affiliated tertiary‐care hospital located in Seoul, South Korea.^25^, ^26^ From the SNUH CDM, we collected data on drug prescriptions, surgical treatments, and diagnostic procedures for each patient. Additionally, we extracted data on RT procedures from SUPREME®.

This study was reviewed and approved by the SNUH Institutional Review Board (IRB). The IRB waived the requirement for obtaining informed consent from the participants (IRB No: C‐2105‐213‐1226). Information that may identify patients was anonymized.

### 2.2 Study population

Eligible patients were those who had been diagnosed with BC, colon cancer (CC), liver cancer (hepatocellular cancer, HC), lung cancer (LC), or prostate cancer (PC), and visited SNUH at least once between January 2014 and December 2019. We selected these cancer types based on their high incidence, prevalence, and the growing financial burdens of their treatments in South Korea.^27^ Patients with cancer were identified using the following diagnosis terms: “cancer,” “carcinoma,” “tumor,” and “malignant”. Any patients diagnosed with benign, borderline, and/or in situ cancer were excluded from the analysis.

### 2.3 Medical care utilization

Medical care utilization by patients with cancer in anticancer agents, RT, surgery, and diagnostic procedures was investigated. We selected anticancer agents from the list of all drugs prescribed to patients with cancer identified in the SNUH CDM. Selected anticancer agents were classified into four groups: cytotoxic agents, hormonal agents, targeted · immune‐oncology agents (e.g., nivolumab and pembrolizumab), and immunomodulators (i.e., immunosuppressants and immune enhancers such as granulocyte‐colony stimulating factor [G‐CSF]). Anticancer agents, which were not listed in the SNUH CDM at the time of conducting this study (e.g., talazoparib and ponatinib), were excluded from the analysis.

RT was classified into five types: photon beam RT (e.g., 10MV X‐ray 2 ports), proton beam RT, three‐dimensional conformal RT (3D‐RT), stereotactic RT (i.e., stereotactic ablative RT [SABR] and fractionated stereotactic RT [FSRT]), and IMRT. We selected the most performed surgical treatment in each cancer type (Table S1) from the list of all surgical treatment operated to patients with cancer identified in the SNUH CDM. Surgeries operated on <0.5% of participants and not directly pertinent to cancer treatment were excluded from the analysis. Likewise, diagnostic procedures were categorized into six types: biopsy, computed tomography (CT), magnetic resonance imaging (MRI), positron emission tomography (PET), ultrasonography and X‐Ray.

### 2.4 Statistical analyses

We identified the number of patients with cancer who received ≥1 anticancer agent, RT, surgery, and diagnostic procedure, respectively, by year and/or type. We examined the prescription proportions of drug class and the proportions of patients who underwent each RT type, surgical treatment, and diagnostic procedure, respectively, by year. We summarized trends by calculating compound adjusted growth rates (CAGR) over the period. CAGR offers a representation of the mean annual growth rate over a specified duration, accounting for compounding across multiple time periods^28^:

$$\text{CAGR}\left(\%\right)=\left[{\left(\frac{\text{Ending value}}{\text{Begining value}}\right)}^{\frac{1}{\text{Number of year}}}-1\right]\times 100$$

Data management, statistical analyses and data visualizations were performed with PostgreSQL (PostgreSQL Global Development Group) with DBeaver (ver 21.0.0) and R software (ver 4.1.3; R Foundation, Vienna, Austria).

## 3 RESULTS

### 3.1 Study population and overall trend

**FIGURE 1:**
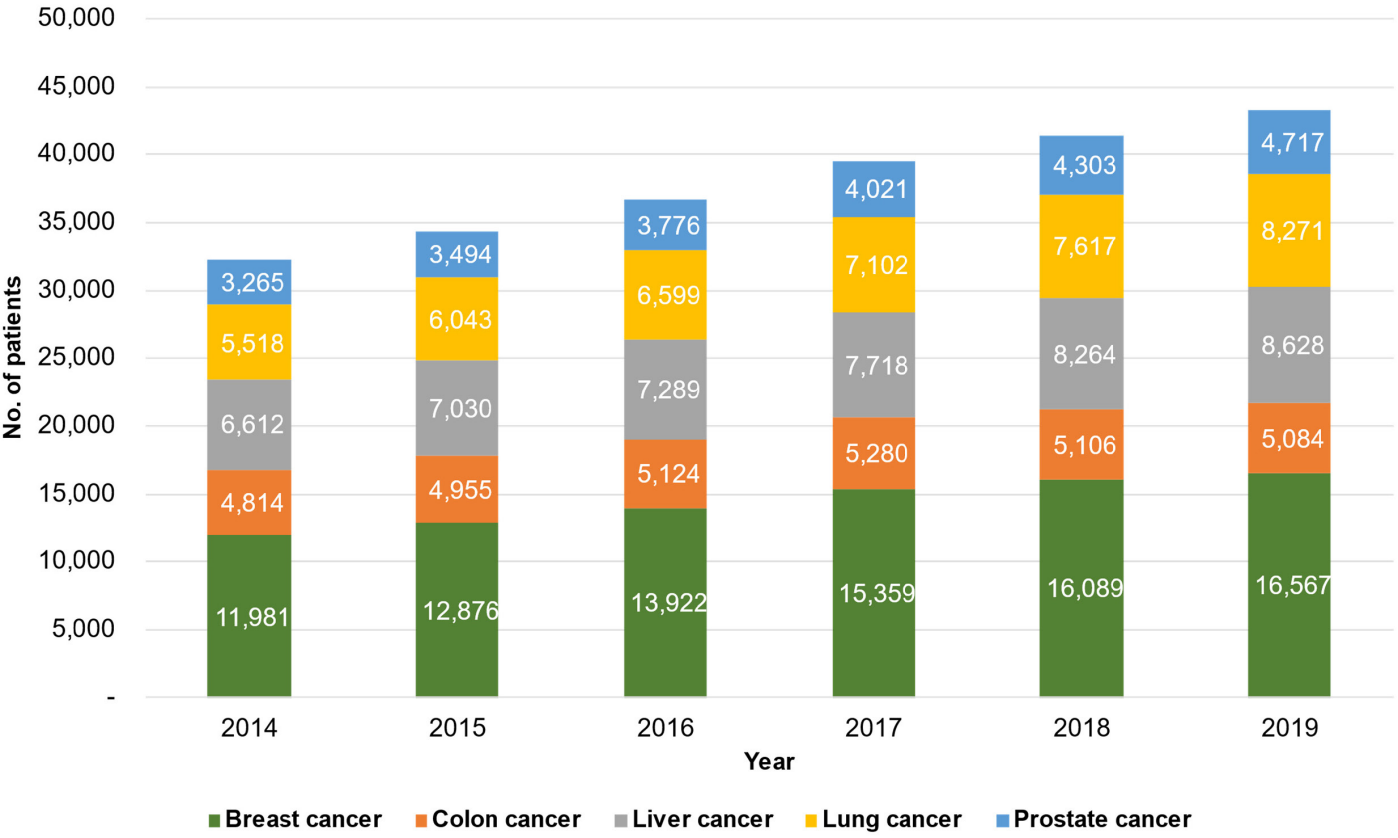
Annual trends in the number of patients with cancer by cancer types. Breast cancer (green), colon cancer (orange), liver cancer (gray), lung cancer (yellow), and prostate cancer (blue).

**FIGURE 2:**
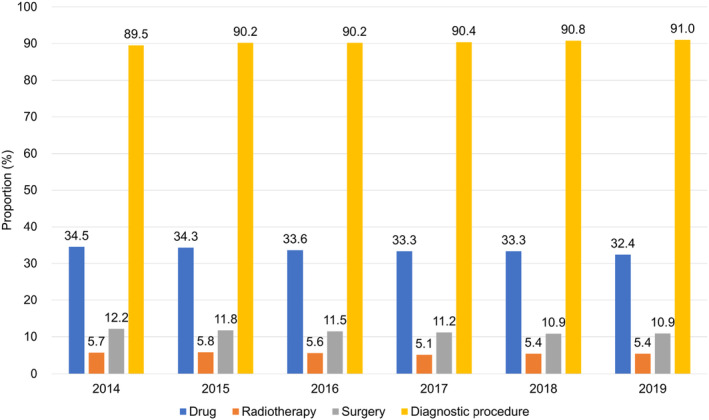
Annual trends in the medical care utilization by patients with cancer for anticancer agents, radiotherapy, surgery, and diagnostic procedures between 2014 and 2019. The bar graphs indicate the proportions of patients with cancer who received ≥1 treatment or diagnostic modality: drug (blue), radiotherapy (orange), surgery (gray), diagnostic procedure (yellow).

A total of 74,285 patients with cancer were identified, of whom 25,793 (34.7%), 10,254 (13.8%), 17,136 (23.1%), 17,423 (23.5%), 7490 (10.1%) were diagnosed with BC, CC, HC, LC, or PC respectively (Table 1). Over the entire period, 29,962 patients (40.3%) received ≥1 anticancer agent, 10,577 patients (14.2%) underwent RT, 23,066 patients (31.1%) had surgery, and 68,849 patients (92.6%) underwent diagnostic procedure. The number of patients with cancer who visited SNUH consistently increased every year from 32,190 in 2014 to 43,267 in 2019 (Figure 1). The proportion of patients with cancer who received ≥1 anticancer agent, RT, and diagnostic procedure have remained relatively stable over the period (CAGR, −1.2%, −1.1%, 0.3%, respectively), whereas the proportion of patients underwent surgical treatment decreased continuously from 12.2% (2014) to 10.9% (2019; CAGR, −2.2%, Figure 2).

**TABLE 1:** Baseline characteristics of eligible patients by cancer type and treatment/diagnostic modality.

	Drug	Radiotherapy	Surgery	Diagnostic procedure
Total (*N* = 74,285)				
No. of patients (%)	29,962 (40.3%)	10,577 (14.2%)	23,066 (31.1%)	68,849 (92.6%)
Male	11,633 (37.6%)	3334 (31.5%)	8805 (38.1%)	30,897 (44.9%)
Female	18,692 (62.4%)	7243 (68.5%)	14,261 (61.9%)	37,952 (55.1%)
Age	57.9 ± 12.2	56.3 ± 12.4	57.4 ± 12.5	58.4 ± 12.0
Breast cancer (*N* = 25,793)				
No. of patients (%)	14,563 (56.5%)	6217 (24.1%)	9944 (38.6%)	24,583 (95.3%)
Male	45 (0.3%)	8 (0.1%)	26 (0.2%)	85 (0.3%)
Female	14,518 (99.7%)	6209 (99.9%)	9918 (99.8%)	24,498 (99.7%)
Age	52.1 ± 10.4	51.5 ± 10.0	51.1 ± 10.5	53.1 ± 10.4
Colon cancer (*N* = 10,254)				
No. of patients (%)	3350 (32.7%)	239 (2.3%)	4931 (48.0%)	9703 (94.6%)
Male	1976 (59.0%)	145 (60.7%)	3003 (60.9%)	5773 (59.5%)
Female	1374 (41.0%)	94 (39.3%)	1928 (39.1%)	3930 (40.5%)
Age	61.8 ± 11.1	63.8 ± 10.9	65.2 ± 11.0	64.2 ± 11.0
Liver cancer (*N* = 17,136)				
No. of patients (%)	4594 (26.8%)	1049 (6.1%)	1932 (11.3%)	16,424 (95.8%)
Male	3466 (75.4%)	853 (81.3%)	1437 (74.4%)	11,384 (66.4%)
Female	1128 (24.6%)	196 (18.7%)	495 (25.6%)	5040 (29.4%)
Age	60.3 ± 10.0	62.3 ± 10.6	58.7 ± 10.9	61.5 ± 10.5
Lung cancer (*N* = 17,423)				
No. of patients (%)	6049 (34.7%)	2578 (14.8%)	4723 (27.1%)	16,476 (94.6%)
Male	3835 (63.4%)	1759 (68.2%)	2580 (54.6%)	9835 (59.7%)
Female	2214 (36.6%)	819 (31.8%)	2143 (45.4%)	6641 (40.3%)
Age	64.1 ± 11.0	66.2 ± 10.9	62.9 ± 11.4	63.6 ± 12.0
Prostate cancer (*N* = 7490)				
No. of patients (%)	2428 (32.4%)	680 (9.1%)	2037 (27.2%)	6321 (84.3%)
Male	2428 (100%)	680 (100%)	2037 (100%)	6321 (100%)
Female	0 (0)	0 (0)	0 (0)	0 (0)
Age	72.4 ± 8.2	71.6 ± 7.7	67.7 ± 6.7	71.7 ± 7.8

*Note*: Values are presented as frequency (%) and age was presented as mean ± standard deviation (years).

### 3.2 Drug use (Figure 3)

**FIGURE 3:**
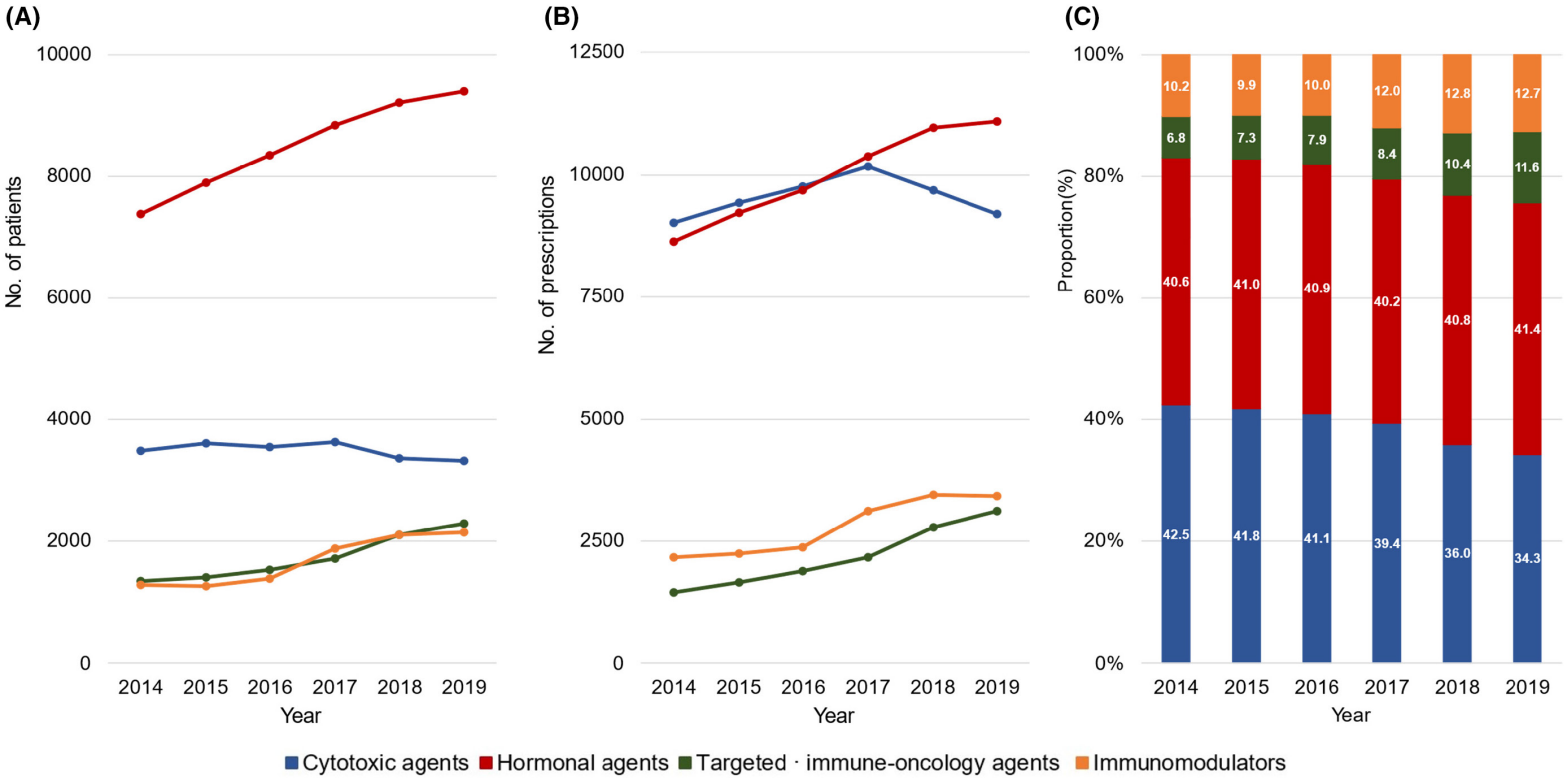
Annual trends in the use of anticancer agents by patients with breast, colon, liver, lung, or prostate cancer between 2014 and 2019: cytotoxic agents (blue), hormonal agents (red), targeted · immune‐oncology agents (green), and immunomodulators (orange). (A) Number of patients prescribed ≥1 anticancer agent by drug class. (B) Number of anticancer agent prescriptions by drug class. (C) Prescription proportions of each drug class.

The use of targeted · immune‐oncology agents increased the most over the period among the four drug classes (number of patients, from 1353 to 2289 [CAGR, 11.1%]; prescription proportion, from 6.8% to 11.6% [CAGR, 11.3%]), followed by immunomodulators (number of patients, from 1279 to 2147 [CAGR, 10.9%]; prescription proportion, from 10.16% to 12.74% from [CAGR, 4.6%]). In contrast, use of cytotoxic agents had continuously decreased over the study period (prescription proportion, from 42.5% to 34.3% [CAGR, −4.2%]), while hormonal agents did not show any noticeable change in prescriptions from 2014 to 2019 (prescription proportion, from 40.6% to 41.4% [CAGR, 0.4%]).

### 3.3 Radiotherapy (Figure 4)

**FIGURE 4:**
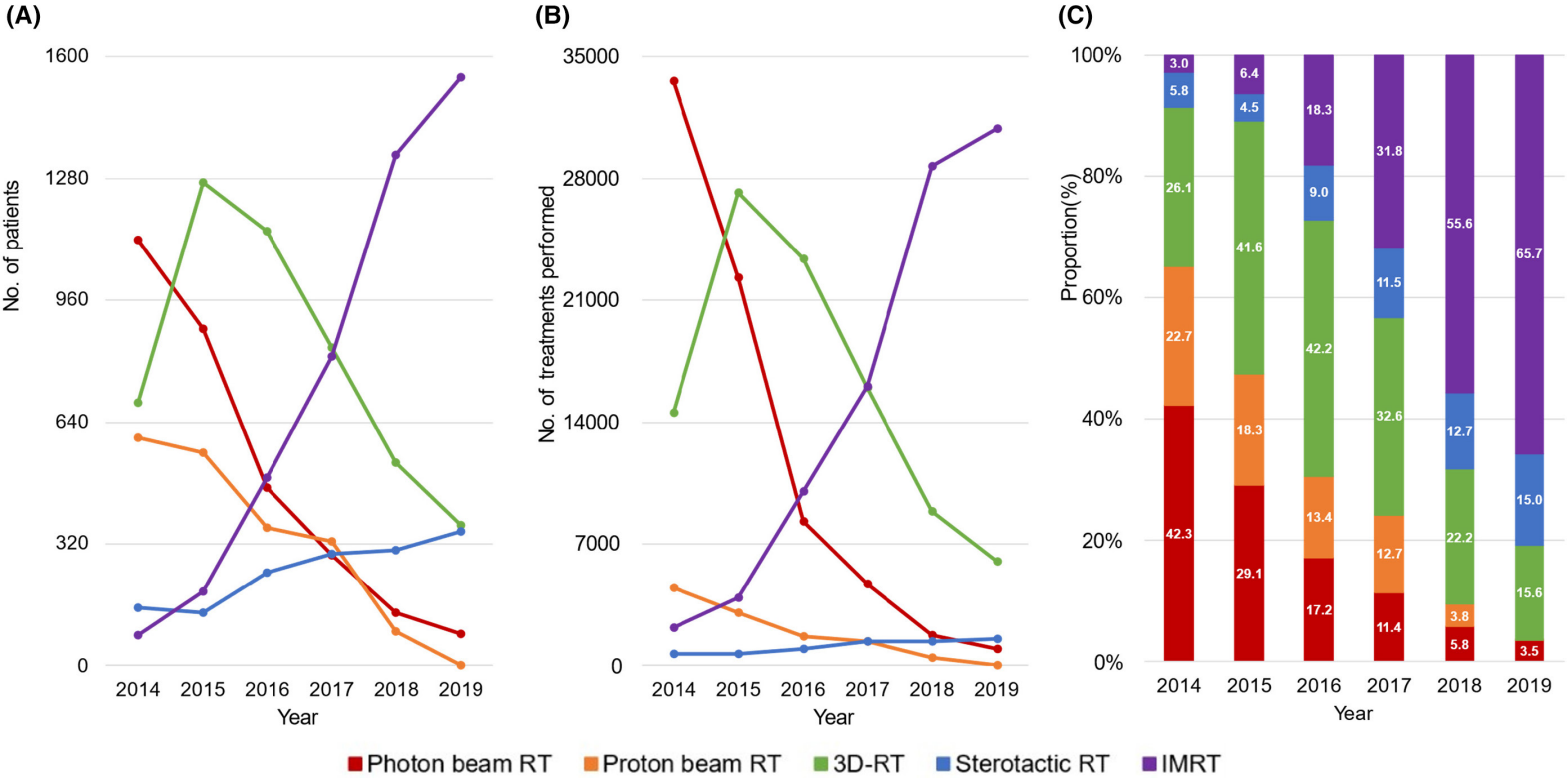
Annual trends in the utilization of RT by patients with breast, colon, liver, lung, or prostate cancer between 2014 and 2019: photon beam RT (red), proton beam RT (orange), 3D‐RT (green), stereotactic RT (blue), and IMRT (purple). (A) Number of patients received ≥1 RT by RT type. (B) Number of treatments performed by RT type. (C) Proportions of RT type. RT, radiotherapy; three‐dimensional conformal RT, 3D‐RT; intensity‐modulated RT, IMRT.

From 2014 to 2019, the use of proton beam RT and photon beam RT dramatically decreased, that is, from 22.7% to 0.1% [CAGR, −64.5%]) and from 42.3% to 3.5% [CAGR, −39.2%], respectively. In contrast, the utilization of IMRT dramatically increased (number of patients, from 80 to 1545 [CAGR, 80.8%]; proportion, from 3.0% to 65.7% [CAGR, 85.0%]), followed by stereotactic RT (number of patients, from 152 to 353 [CAGR, 18.4%]; proportion, from 5.8% to 15.0% [CAGR, 21.1%]). On the other hand, the use of 3D‐RT nearly doubled from 2014 to 2015 (number of patients, from 690 to 1269; proportion, 26.1% to 42.2%), then steadily decreased since 2015.

### 3.4 Surgery (Figure 5)

**FIGURE 5:**
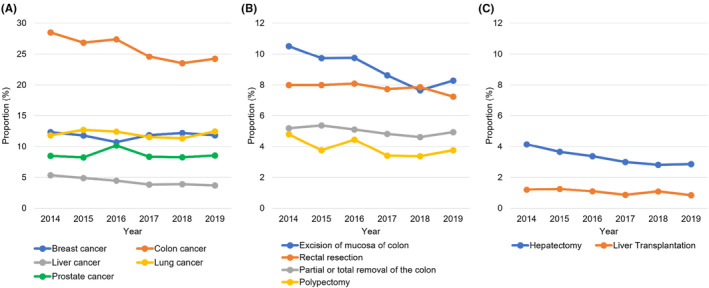
Annual trends in the utilization of surgical procedures by patients with breast, colon, liver, lung, or prostate cancer between 2014 and 2019. (A) Proportions of patients who received surgical treatments by year and cancer types: breast cancer (blue), colon cancer (orange), liver cancer (gray), lung cancer (yellow), and prostate cancer (green). (B) Proportions of patients who received each surgical procedure by year in colon cancer: excision of mucosa of colon (blue), rectal resection (orange), partial of total removal of colon (gray), polypectomy (yellow). (C) Proportions of patients who received each surgical procedure by year in liver cancer: hepatectomy (blue) and liver transplantation (orange).

The most commonly performed surgical procedures were breast‐conserving surgery (annual proportion of patients who underwent the surgery, 8.5 ± 0.6%), excision of mucosa of colon (9.1 ± 1.1%), hepatectomy (3.3 ± 0.5%), pulmonary resection (12.1 ± 0.6%), and prostatectomy (8.7 ± 0.7%) in BC, CC, HC, LC, and PC, respectively (Table S1). There was no significant change in surgical trends over the period, including the types of surgical procedures performed by patients with cancer and the percentage of each surgical procedure except for CC and HC. The proportion of CC patients and HC patients underwent surgical treatment decreased from 28.5% in 2014 to 24.2% in 2019 (CAGR, −3.2%), and from 4.1% in 2014 to 2.9% in 2019, (CAGR, −7.1%), respectively.

### 3.5 Diagnostic procedure (Figure 6)

**FIGURE 6:**
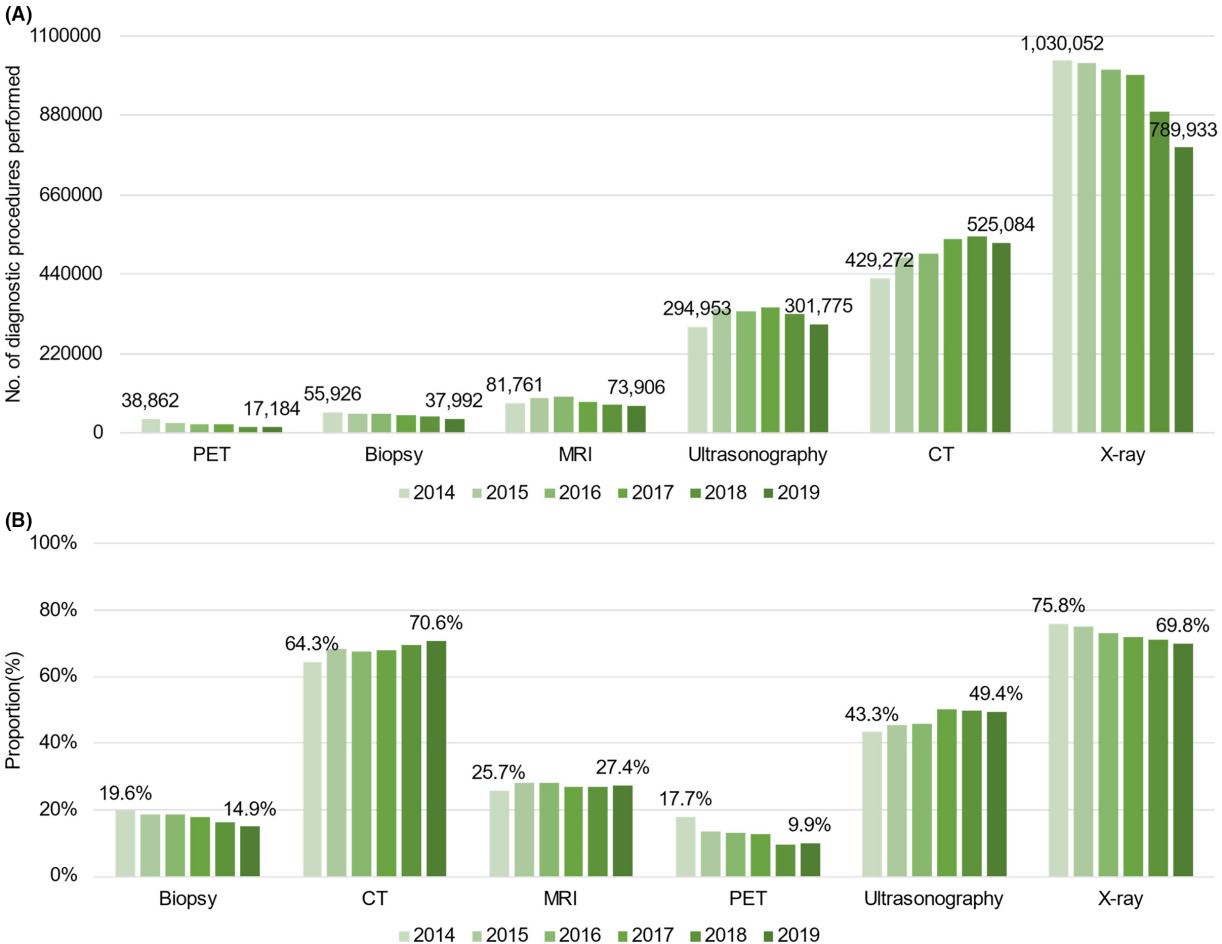
Annual trends in the utilization of diagnostic procedures by patients with breast, colon, liver, lung, or prostate cancer between 2014 and 2019. (A) Number of diagnostic procedures performed by modality and year. (B) Proportions of patients who received each diagnostic procedure by year. CT, computed tomography; MRI, magnetic resonance imaging; PET, positron emission tomography.

X‐ray was the most performed diagnostic procedure while the proportion of patients undergoing X‐ray kept decreasing over the period from 75.8% in 2014 to 69.8% in 2019 (CAGR, −1.6%). In contrast, the proportions of patients undergoing ultrasonography, CT, and MRI increased over the period (CAGR, 2.7%, 1.9%, and 1.3%, respectively). All in all, PET, biopsy and MRI were used least among the diagnostic procedures.

## 4 DISCUSSION

We observed a 1.7‐fold increase in the use of targeted · immune‐oncology agents (from 6.8% to 11.6%, Figure 3), and a 21‐fold increase (from 3.0% in 2014 to 65.7%, Figure 4) in IMRT use from 2014 to 2019. These findings were consistent with the results in previous studies, reporting a 1.7‐ and 1.6‐fold increase in the use of antineoplastic monoclonal antibodies and protein kinase inhibitors, respectively, from 2010 to 2016,^29^, ^30^ as well as an 18‐fold increase in IMRT utilization from 2011 to 2018 in Korea.^17^ The increased use of targeted · immune‐oncology agents and IMRT in patients with cancer is most likely attributed to the fact that their clinical utility has been repeatedly shown. For example, targeted · immune‐oncology agents improved treatment outcomes in patients with cancer although the extent of their efficacy varied depending on the specific cancer type and treatment setting (e.g., lines of therapy).^31^, ^32^, ^33^ Likewise, several studies have demonstrated the effectiveness of IMRT in improving overall survival and reducing radiation‐induced toxicity compared with conventional radiation therapy techniques such as 2D and 3D‐RT.^34^, ^35^, ^36^, ^37^

In contrast, we observed a continuous decrease in the proportion of patients who underwent surgical treatment, from 12.2% in 2014 to 10.9% in 2019 (CAGR, −2.2%, Figure 2). This decrease was particularly noticeable in patients with CC (from 28.5% to 24.2% [CAGR, −3.2%]) and HC (from 4.1% to 2.9% [CAGR, −7.1%], Figure 5). The decline in surgical treatment may have been caused by increased adoption of non‐surgical treatments, possibly driven by advancements and wider use of targeted · immune‐oncology agents.^38^, ^39^ To support this notion, a substantial number of targeted · immune‐oncology agents, including multikinase inhibitors (e.g., regorafenib, lenvatinib, cabozantinib, and ramucirumab) and immune checkpoint inhibitors (e.g., nivolumab and pembrolizumab), were approved by the regulatory agencies for the treatment of CC and HC between 2015 and 2019.^32^, ^33^, ^34^, ^35^

The targeted · immune‐oncology agents have undeniably brought new hope to patients with cancer.^31^, ^32^, ^33^, ^40^ Many countries, including South Korea, have leveraged their national health insurance systems and initiated support programs, such as the Financial Aid Program for Cancer Patient (FAPCP), to enhance patient accessibility to these treatments.^41^, ^42^, ^43^, ^44^ We observed that such programs have indeed amplified the usage of the targeted · immune‐oncology agent (Figure 3).^29^, ^30^ Despite these efforts in South Korea, many individuals have not benefited from these programs due to the complexities and limitations of insurance coverage for such treatments.^8^, ^11^, ^12^ Moreover, the growing popularity of targeted · immune‐oncology agents may have resulted in a financial burden on patients and healthcare systems because they are expensive and the long‐term sustainability of their use is uncertain.^2^, ^45^, ^46^, ^47^, ^48^ Thus, we highlight that further studies are necessary to evaluate the cost‐effectiveness of targeted · immune‐oncology agents to ensure long‐term affordable and sustainable patient access.

Over the past few years, the adoption of IMRT has raised globally.^17^, ^49^ While the potential benefits of IMRT are manifold, its widespread use demands rigorous oversight, as the American Society for Radiation Oncology (ASTRO) has cautioned against IMRT's inappropriate utilization and potential overuse, especially in cases where IMRT may not provide significant clinical advantages.^13^, ^34^, ^35^, ^36^ In South Korea, the expansion of health insurance coverage for IMRT since 2015 may have led to overutilization of IMRT.^17^ Indeed, we observed a sharp increase of IMRT use since 2015 (Figure 4). We emphasize the necessity to implement quality control measures to ensure appropriate use and prevent the misuse and abuse of IMRT.

This study had three major limitations. First, this study's results may not be generalizable to other hospital settings because the analysis was based on data from a single institution. However, the consistency of our findings with those from other studies may support that the results are practically extrapolated to other situations. Second, some anticancer agents and procedures were not included in the analysis because they were not available in the SNUH CDM at the time of the study. This may have led to an underestimation of the actual number of patients who received each treatment or diagnostic procedure. Third, we were unable to segregate the patient cohort into more granular subcategories and perform advanced statistical analysis such as time series analysis with diverse parameters. This limitation is inherent to the retrospective observational study and the available data variables. For example, patient phenotyping was problematic, as we could not discern between those newly diagnosed and returning patients; the original diagnosis location for patients at SNUH, especially if they had prior diagnoses elsewhere, was unclear.

Despite these limitations, to the best of our knowledge, our study is the most up‐to‐date and exhaustive analysis in South Korea which have included treatments and diagnostic procedures that are newly developed and not yet covered by insurance. We successfully achieved our primary objective of illustrating the current trends. However, more intricate analyses, such as detailed stratification of patient with richer datasets and enhanced time series analysis, could potentially reveal trends and patterns not identified in our study.

In conclusion, the use of targeted · immune‐oncology agents and IMRT had continuously increased over the period of 2014–2019. Therefore, healthcare policymakers and practitioners should focus on the evaluation of long‐term cost‐effectiveness of targeted · immune‐oncology agents and the implementation of quality control measures for IMRT.

## AUTHOR CONTRIBUTIONS

**Jung‐Hyun Won:** Conceptualization (equal); data curation (lead); formal analysis (lead); investigation (lead); methodology (equal); software (equal); visualization (lead); writing – original draft (lead). **Tae Kyu Chung:** Conceptualization (equal); data curation (equal); methodology (equal); software (equal); validation (equal); writing – review and editing (equal). **Joochul Lee:** Conceptualization (supporting); software (supporting). **Sangwon Yoon:** Conceptualization (supporting); funding acquisition (equal); resources (equal). **Yoomin Jeon:** Conceptualization (equal); data curation (equal); funding acquisition (equal); methodology (equal); project administration (equal); resources (equal); software (equal); supervision (equal); validation (equal); writing – review and editing (equal). **Howard Lee:** Conceptualization (equal); funding acquisition (equal); methodology (equal); project administration (lead); resources (equal); supervision (lead); validation (equal); writing – review and editing (lead).

## FUNDING INFORMATION

This research was funded by SELVAS AI Inc. However, the sponsors of the study played no part in the design, data collection, data analysis, and data interpretation. Additionally, the BK21FOUR Program of the National Research Foundation of Korea (NRF), funded by the Ministry of Education (5120200513755), provided additional support for this research.

## CONFLICT OF INTEREST STATEMENT

All authors declared no competing interests for this work.

## ETHICS STATEMENT

This study was reviewed and approved by the Seoul National University Hospital Institutional Review Board (IRB). The IRB waived the requirement for obtaining informed consent from the participants (IRB No: C‐2105‐213‐1226). Information that may identify patients was anonymized.

## Supporting information


**Supplementary Table 1.** List of most performed surgical treatment and the annual proportion of patients with cancer who underwent the surgery in each cancer type.

## Data Availability

The data supporting the findings of this study are available from the corresponding author upon reasonable request, in accordance with the guidelines set forth by the Seoul National University Hospital Institutional Review Board.
