# Supplementary material for: Trends in medical care utilization in patients with cancer: An analysis of real‐world data in a tertiary hospital in Korea, 2014–2019
Source: Cancer Med. 2023 Oct 30;12(22):21022–31. doi: 10.1002/cam4.6660 (PMC10709731; doi:10.1002/cam4.6660)
Supplement: Supplementary file 1 — Supplementary Table 1. List of most performed surgical treatment and the annual proportion of patients with cancer who underwent the surgery in each cancer type. [file CAM4-12-21022-s001.docx]

# SUPPLEMENTARY

**Supplementary Table 1.** List of most performed surgical treatment and the annual proportion of patients with cancer who underwent the surgery in each cancer type.

|  | **Surgical treatment**  **(Annual proportion of cancer patients who underwent the surgery)** | **Surgical treatment terms in SNUH CDM** |
| --- | --- | --- |
| **Breast cancer** | Breast-conserving surgery (8.5±0.6%) | Ultrasonography guided vacuum assisted excision of breast, quadrantectomy of breast, partial mastectomy, mammoplasty, lumpectomy of breast |
|  | Mastectomy (2.5±0.2%) | Simple mastectomy |
|  | Breast reconstruction surgery (0.8±0.3%) | Reconstruction of breast using free transverse rectus abdominis myocutaneous flap, insertion of prosthesis for breast |
| **Colon cancer** | Excision of mucosa of colon (9.1±1.1%) | Colonoscopy and excision of mucosa of colon, endoscopic excision of lesion of large intestine |
|  | Rectal resection (7.8±0.3%) | Anterior resection of rectum, laparoscopic-assisted anterior resection of rectum, low anterior resection of rectum, sigmoidoscopic excision of mucosa of rectum, hartmann operation, rectal resection |
|  | Partial or total removal of the colon (5.0±0.3%) | right colectomy, laparoscopic right hemicolectomy, extended right hemicolectomy, left colectomy, laparoscopic left hemicolectomy, partial resection of colon, total colectomy |
|  | Polypectomy (3.9±0.6%) | Colonoscopic polypectomy, endoscopic excision of polyp of large intestine using sigmoidoscope |
| **Liver cancer** | Hepatectomy (3.3±0.5%) | Resection of segment of liver, lobectomy of liver, excision of lesion of liver, liver excision, extended right hemihepatectomy |
|  | Liver transplantation (1.1±0.2%) | Transplantation of liver |
| **Lung cancer** | Pulmonary resection (12.1±0.6%) | Lobectomy, lobectomy of lung, partial lobectomy of lung, excision of segment of lung, wedge excision of lung, wedge resection, total pneumonectomy, bilobectomy of lung, pneumonectomy with tracheobronchoplasty, lung excision, excision of segment of right upper lobe |
| **Prostate cancer** | Prostatectomy (8.7±0.7%) | Radical retropubic prostatectomy, robot assisted laparoscopic radical prostatectomy, transurethral prostatectomy, retropubic prostatectomy, radical prostatectomy |
